# Cremophor EL Nano-Emulsion Monomerizes Chlorophyll *a* in Water Medium

**DOI:** 10.3390/biom9120881

**Published:** 2019-12-16

**Authors:** Ewa Janik-Zabrotowicz, Marta Arczewska, Monika Zubik, Konrad Terpilowski, Tomasz H. Skrzypek, Izabela Swietlicka, Mariusz Gagos

**Affiliations:** 1 Department of Cell Biology, Institute of Biological Sciences, Maria Curie-Sklodowska University, ul. Akademicka 19, 20-033 Lublin, Poland; mariusz.gagos@poczta.umcs.lublin.pl; 2 Department of Biophysics, University of Life Sciences in Lublin, Akademicka 13, 20–950 Lublin, Poland; marta.arczewska@up.lublin.pl (M.A.); izabela.swietlicka@up.lublin.pl (I.S.); 3 Department of Biophysics, Institute of Physics, Maria Curie-Sklodowska University, Radziszewskiego 10, 20–031 Lublin, Poland; monika.zubik@gmail.com; 4 Department of Physical Chemistry-Interfacial Phenomena, Maria Curie-Sklodowska University, Pl. Marii Curie-Sklodowskiej 3, 20–031 Lublin, Poland; terpil@poczta.umcs.lublin.pl; 5 Laboratory of Confocal and Electron Microscopy, Department of Biotechnology and Environment Sciences Center for Interdisciplinary Research, John Paul II Catholic University of Lublin, ul. Konstantynów 1J, 20–708 Lublin, Poland; tomasz.skrzypek@kul.pl

**Keywords:** chlorophyll *a*, Cremophor EL, nano-emulsion, monomer, molecular spectroscopy

## Abstract

In this paper, the application of a non-ionic detergent Cremophor EL for monomerization of chlorophyll *a* in an aqueous medium is studied. The spectrophotometric properties of chlorophyll *a* encapsulated into the Cremophor EL nano-emulsion system were characterized by electronic absorption, steady-state and time-resolved fluorescence as well as circular dichroism spectroscopy. The results have shown that chlorophyll *a* dissolves more efficiently in the aqueous medium containing low-level Cremophor (5 wt%) than at an ethanolic solution even in the concentration of 10^−4^ M. The molecular organization of the chlorophyll *a* in the Cremophor EL nano-micelles was also investigated by means of Raman spectroscopy. The spectral changes in the frequency of the C=O stretching group were used to distinguish the aggregation state of chlorophyll. It was revealed that chlorophyll *a* exists dominantly in the monomeric form in the Cremophor EL aqueous solution. The promising aspect of the use of Cremophor EL nano-emulsion as a delivery system is to maintain stable chlorophyll monomer in an aqueous medium. It would open the potential for a new, practical application of chlorophyll *a* in medicine, as a dietary supplement or studies on molecular organization of chlorophyll *a* in the well-defined artificial system.

## 1. Introduction

There are few biomolecules related to their chemical structure investigation that have been mentioned by the laureates in the Nobel award lectures [1,2,3]. Among them, chlorophylls (chls), which are largely involved in the biological process such as photosynthesis, is often referred to be “the pigments of life” [4].

Chlorophylls (chls) are the most abundant class of biomolecules present in the world. Chls are becoming more and more popular due to their multiple bioactive properties. As evidenced by a number studies, they are much more than just green pigments found in the photosynthetic organisms. Their extensive use in the pharmaceutical and food industries was reported [5,6]. Although biomedical applications illustrate the use of chlorophyll as a photosensitizer in the photodynamic therapy (PDT), the recent research works being of significant interest are also focused on its potential role as an anti-cancer agent [7,8,9,10] with antigeno-toxic properties [11], and antioxidant capacity to scavenge free radicals and to prevent lipid oxidation [12].

Chemically, chls belong to a class of porphyrins and are composed of a tetrapyrrole moiety with an Mg^2+^ ion coordinated to nitrogens. Additionally, chls have one reduced pyrrole ring and a long non-polar phytyl chain (except chl *c*) [13]. Due to the hydrophobic character of molecular structure, chls have a tendency to aggregate in aqueous media even in the micromolar concentration range [14,15]. It is known that a good candidate for a drug formulation should be prepared in aqueous solution to provide high biocompatibility and bioavailability, and maintaining low self-aggregation of chls is highly desirable [16]. There are few commercial formulations with beneficial effects on health which are based on chlorophyll-related compounds; however, most of them do not contain the chemical compound chlorophyll, but rather derivatives that are called chlorophyllin. Sodium/copper chlorophyllin is the widely used copper-chlorophyll derivative. It is a water-soluble copper complex of hydrolyzed chlorophylls that exhibits potent antimutagenic and antioxidant activities [11,17].

**Scheme 1:**
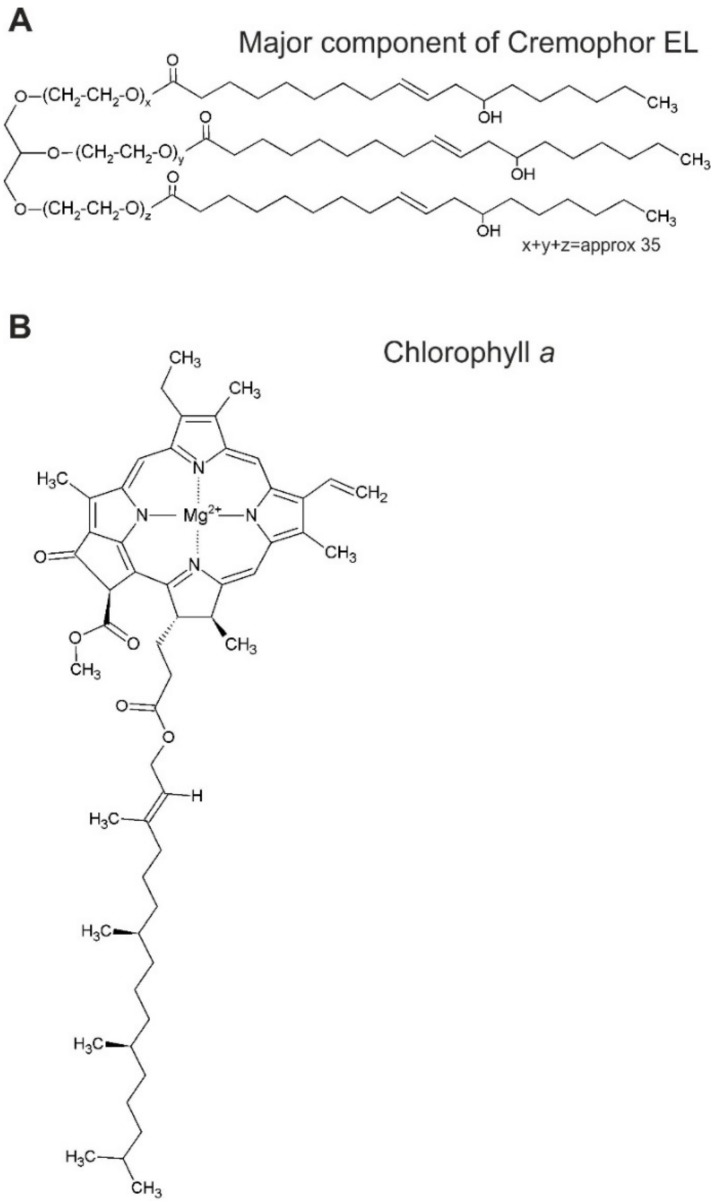
The chemical structure of Cremophor EL (polyoxyethyleneglycerol triricinoleate 35, panel **A**) and chlorophyll *a* (panel **B**). x, y, z represent the number of oxyethylen units in each chain.

By now, over a hundred chlorophyll-type pigments are known, but the most widely distributed form is chlorophyll *a* (chl *a*), which, in higher plants, is accompanied at a 3:1 ratio by chlorophyll *b* (having a formyl group in its structure instead of a 7-methyl group) [18], see Scheme 1. In vivo, the main role played by the chl *a* molecule is the collection of the solar energy. In the plant cell, molecules of chl *a* are embedded in the protein bed of the photosynthetic complexes named photosystems I and II located in the thylakoid membranes of chloroplast. It was evidenced that the chl *a* function results from its molecular organization induced by the supramolecular structure of the photosynthetic pigment-protein complexes, e.g., chl *a* molecules of photosystem II core complex or trimeric LHCII (light-harvesting complex of photosystem II) are responsible for efficient energy transfer, but a special pair of chls in dimeric LHCII contributes to energy excess quenching [19,20,21]. Likewise, molecular organization of isolated chl *a* molecules determines its ability for energy transfer (monomers) or energy quenching (dimers and aggregates) [22].

Chl *a* is not soluble in water, and for several years, many attempts were made to solubilize it in aqueous media to be attractive for medical application. The previous studies have shown that chl *a* was effectively solubilized in the aqueous medium by means of various cyclodextrins (CDs) and indicated the formation of host–guest inclusion complexes between chl *a* and CDs [23,24]. More recently, chl *a* was blocked inside the chitosan films, and cetyltrimethylammonium chloride was chosen to solubilize it in the water medium with gold nanoparticles [25,26]. However, despite numerous research results on chl *a* organization and application, it should be borne in mind that, as was advised by Hans Fischer during his Nobel lecture, ‘the chlorophyll molecule still presents many riddles’.

Nano-emulsions have attracted a lot of attention in the biomedical, chemical and physical fields due to a remarkable small droplet size and thermodynamicall stability, regardless of the preparation method. Encapsulation of chl *a* in the nano-emulsion composed of a surfactant and water seems to be essential for disclosing some properties of natural photosynthetic structures in vitro, e.g., electron and excitation-energy transport by chl with a well-defined organization. On the other hand, the results will be further used during the development of formulations of chl-based photosensitizers for the use in the photodynamic and anticancer therapies. The study discussed below may also provide a promising approach to formulate a nano-emulsion with low levels of surfactant which is of significant importance for commercial delivery systems with chl *a* used in oral administration (e.g., a dietary supplement).

Hence, in this paper, an aqueous mixture with the non-ionic detergent Cremophor EL (CrEL) allowing the delivery of chl *a* molecules in the water system, mainly in the monomeric molecular form was prepared. Chl *a* monomerization has significant importance due to its known photoactivity [26] and expected efficient uptake of such a molecular form. A small molecule tends to penetrate the cell membrane more easily than a larger one. Cremophor EL (polyoxyl 35 castor oil, the chemical structure presented in Scheme 1) is a polyoxyethylated derivative of hydrogenated castor oil that contains about 87% of ricinoleic acid.

It is an oily liquid at 25 °C and has an approximate critical micelle concentration (CMC) at 0.02% [27]. In pharmaceutical applications, the non-ionic surfactant CrEL has been extensively used for encapsulation and solubilization of various hydrophobic bioactive compounds [28]. A good example would be the pharmaceutical formulation of the anticancer agent paclitaxel, which contains 50% CrEL [29,30].

In our study, the spectrophotometric properties of chl *a* encapsulated into the CrEL nano-emulsion system were evaluated using electronic absorption, steady-state and time-resolved fluorescence as well as circular dichroism spectroscopy. The molecular organization of chl *a* in the CrEL nano-micelles was estimated based on Raman spectroscopy as well.

## 2. Materials and Methods

### 2.1. Materials

CrEL (polyoxyl 35 castor oil, MW = 2500 g·mol^−1^) and phosphate buffered saline (PBS, pH 7.4) were purchased from Sigma Aldrich (St. Louis, MO, USA). EtOH (96%) and all other chemicals were of analytical grade.

#### 2.1.1. Chlorophyll *a* Isolation

Chl *a* (MW = 893.5 g·mol^−1^) was isolated from the fresh spinach leaves homogenized with acetone (1:4 (*w*:*v*)) and filtered through Miracloth. Next, the acetone extract was mixed with n-hexane at the ratio 50:1 (*v*:*v*) to concentrate the pigments. The hexane fraction was chromatographed on HPTLC Silica gel 60 plates (Merck, Germany) using the mobile phase of 2-propanol:n-hexane (*v*:*v*, 1:9). After pigments separation adequate layer with chl *a* was transferred into tubes with 100% acetone and centrifuged. Next, the supernatant was evaporated under a nitrogen stream to obtain a solid form of pigment. Chl *a* was identified based on spectral properties.

#### 2.1.2. Chlorophyll *a* in Ethanol or PBS Buffer

Chl *a* in a solid form was dissolved in adequate volumes of EtOH or PBS buffer (pH 7.4). The mixture was slowly stirred at 23 °C for 15 min.

#### 2.1.3. Cremophor Nano-Emulsion Preparation

Firstly, the non-ionic surfactant (CrEL) was dissolved in the co-surfactant (96% EtOH). Next, the mixture was added into an aqueous phase (PBS buffer (pH 7.4)) while slowly stirring at 23 °C for 1 h for spontaneous formation of nano-micelles of CrEL. In the experiment different wt% concentrations of CrEL in the PBS buffer (0.1, 0.2, 0.5, 1, 2, 5, 25, 50, 100 wt%) were used. Instability of cremophor nano-micelles was excluded using the dynamic light scattering method.

#### 2.1.4. Complex Formation with Chlorophyll *a*

Chl *a* was dissolved in 96% EtOH. Next, it was added to the cremophor emulsion, while slowly stirred at 23 °C for 15 min at the ratio 1:40 (*v*:*v*). The final chl *a* molar concentrations in the samples were: 10^−4^ M, 10^−5^ M or 10^−6^ M.

### 2.2. Methods

#### 2.2.1. UV-Vis Absorption Spectroscopy

UV-Vis absorption spectra were measured using the Cary 60 (Agilent Technologies, Santa Clara, California, USA) spectrophotometer in the range of 200–800 nm at room temperature.

#### 2.2.2. Circular Dichroism Spectroscopy

CD spectra were recorded by a Chirascan-plus spectrometer (Applied Photophysics, Leatherhead, UK) in the range of 300–800 nm at room temperature.

#### 2.2.3. Steady-State Fluorescence Spectroscopy

Room temperature fluorescence emission spectra were registered with a F-7000 spectrofluorometer (Hitachi, Hitachi, Japan). 77 K fluorescence emission spectra were recorded with a Cary Eclipse spectrofluorometer (Varian, Sydney, Australia). The excitation was set at 434 nm. The excitation and emission slits were 5 nm.

#### 2.2.4. Time-Resolved Fluorescence Spectroscopy

The fluorescence lifetime was registered using a FluoTime 300 spectrometer (PicoQuant, Berlin, Germany). The excitation was set at 405 nm from a solid state LDH-P-C-405 laser with 20 MHz frequency of pulses and with a pulse width of 70 ps. The emission was detected at 670 nm by a micro-channel plate and the time-correlated single-photon counting system PicoHarp 300. The fluorescence decay curves were fitted with FluoFit Pro v 4.5.3.0 (PicoQuant) software. The fluorescence intensity decays were analyzed by reconvolution with the instrument response function and analyzed as a sum of exponential terms. The quality of the fit was assessed from the χ^2^ value.

#### 2.2.5. Raman Spectroscopy

Raman spectroscopy was performed on the liquid samples placed into glass capillary tubes (sealed on both sides) using an inVia confocal Raman microscope (Renishaw, Gloucestershire, UK) equipped with a 20× long working distance objective (SLM Plan N, NA = 0.25, Olympus, Tokyo, Japan) and with a high sensitivity, ultra-low noise EMCCD (Electron Multiplying Charged Coupled Device) detection camera Newton 970 (Andor, Belfast, UK). The samples were excited with an argon laser (Stellar-REN, Modu-Laser, Centerville, UT, USA) operating at 457 nm (0.35 μW maximum laser power). Spectra were obtained at 1 cm^−1^ resolution (2400 lines/mm grating) in the spectral region of 325–1995 cm^−1^. The total acquisition time was about 50 s. Spectral acquisition and pre-processing were performed with the Renishaw WIRE 4.4 software from Renishaw.

#### 2.2.6. Dynamic Light Scattering

The micelle sizes were determined using Zetasizer Nano ZS90 (Malvern Instruments Ltd., Malvern, UK). The refraction index value was set at 1.33. Zeta potential was calculated according to the Smoluchowski equation.

#### 2.2.7. Cryo-Scanning Electron Microscopy

The morphology of micelles was imaged using the Zeiss Ultra Plus (Carl Zeiss Microscopy GmbH, Jena, Germany) scanning electron microscope, keeping the sample stage at −160 °C. The samples were frozen in liquid nitrogen at −190 °C and next were shadowed with platinum. Observations were made at 3 kV. Images were taken at a magnification of 470,000.

## 3. Results and Discussion

**Figure 1:**
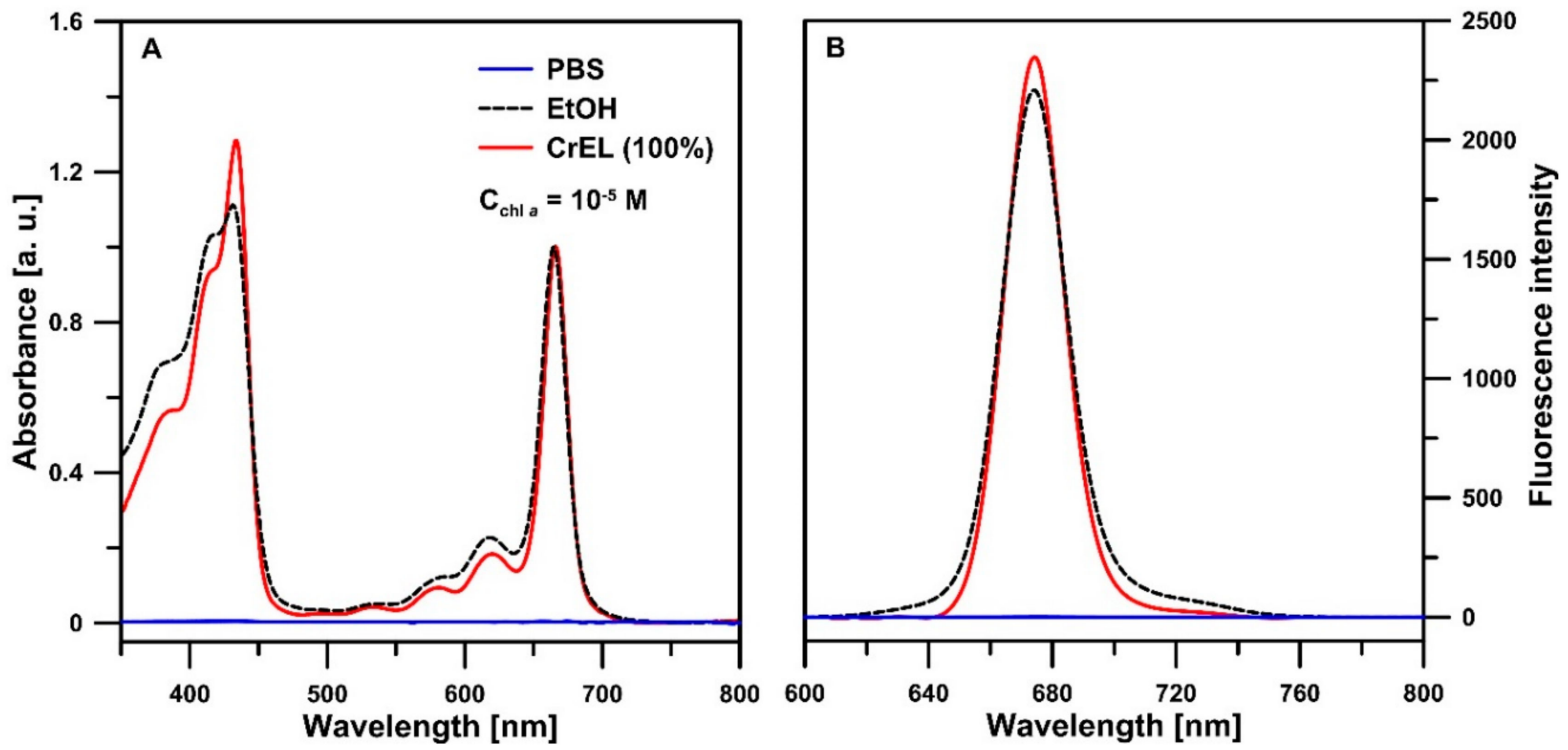
Electronic absorption and fluorescence emission spectra of chlorophyll *a* dissolved in Cremophor EL, ethanol or PBS buffer. Absorption spectra (**A**) and fluorescence emission spectra (**B**) measured from chl *a* dissolved in CrEL (CrEL (100%), red solid line), 96% ethanol (EtOH, black dashed line) or PBS buffer (PBS, blue solid line, the spectra were magnitude 10 times). The molar concentration of chl *a* in the samples was 10^−5^ M. The absorption spectra were normalized in the Q spectral region. The spectra were measured at room temperature.

Figure 1 presents electronic absorption and fluorescence emission spectra (registered at room temperature) of chl *a* (10^−5^ M) dissolved in different solvent media.

**Figure 2:**
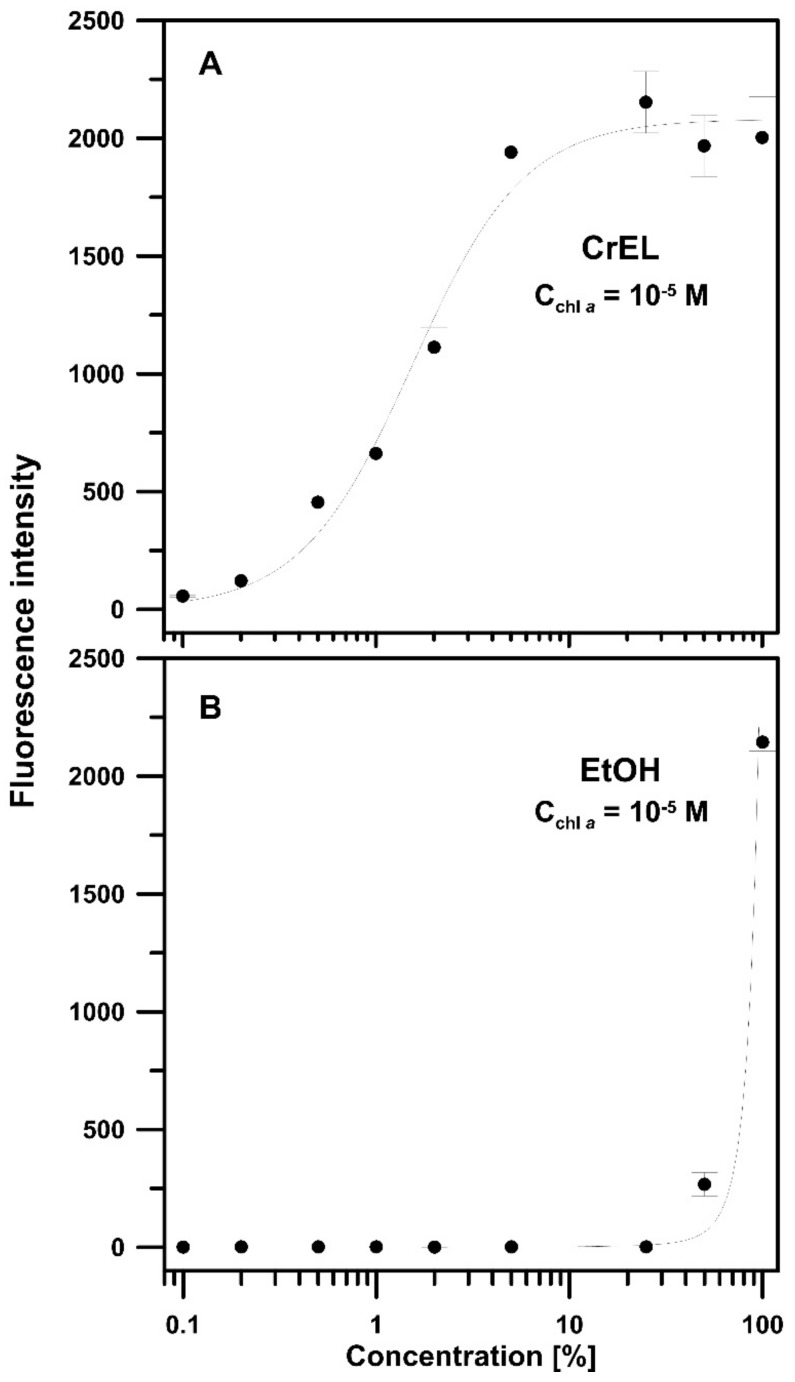
Effect of wt% concentration of Cremophor EL or 96% ethanol in the PBS buffer on the chlorophyll *a* fluorescence emission intensity. The samples were prepared by chl *a* dissolution in 15 µL of 96% EtOH followed by its suspension in the PBS buffer containing CrEL (CrEL, **A**) or EtOH (EtOH, **B**), respectively, in 0.1, 0.2, 0.5, 1, 2, 5, 25, 50, 100 wt% concentration. The molar concentration of chl *a* in the samples was 10^−5^ M. The excitation was at 434 nm. Error bars indicate standard deviation (*n* = 3 biological replicates).

It is known that in water chl *a* displays supramolecular aggregation behavior [31,32]. Due to a lack of solubility of chl *a* in an aqueous medium (in our study—PBS buffer, pH 7.4), this pigment is determined in many organic solvents. Chl *a* is known as a hydrophobic molecule that is efficiently soluble, among others, in acetone, methanol, ethanol, THF, n-hexane, chloroform [15,32,33]. The absorption spectrum of ethanolic solution (Figure 1A) is characterized by the bands centered at 432 nm (Soret spectral region) and 665 nm (Q spectral region) typical for chl *a* monomer [34]. The fluorescence emission spectrum, measured at room temperature, (Figure 1B) with one band located at 675 nm confirms the presence of monomeric form of chl *a* at such pigment concentration in 96% EtOH [15,34,35]. In this study, chl *a* was also dissolved in the non-ionic, amphiphilic detergent known as CrEL. To our knowledge, this detergent was used to study on chl *a* physicochemical properties only once in the past [36]. The absorption (maxima at 434 nm and 666 nm) and fluorescence emission (maximum at 675 nm) spectra of chl *a* in CrEL presented in Figure 1, have the same trace as the spectra of chl *a* in EtOH pointing out to the presence of the pigment monomeric form at the applied concentration of this detergent. According to the literature data, CrEL was repeatedly used to dissolve different biologically active substances insoluble in water [28,37,38]. In our study it has been used for the first time to achieve and stabilize the monomeric form of chl *a* in an aqueous medium. It would open the potential for a new, practical application of the chl *a* molecule in medicine, as a dietary supplement, or studies on molecular organization of chl *a* or the other photosynthetic pigments in the well-defined artificial system. The aim of the presented study was to develop, at low-level CrEL, nano-emulsion, due to: i) development of a stable nano-emulsion [27], ii) potential oral administration of chl *a* in the form of such a system, iii) achievement of the lowest possible value of the CrEL/water ratio. Therefore, different wt% concentrations (0.1, 0.2, 0.5, 1, 2, 5, 25, 50, 100%—on CMC of CrEL) of CrEL as a surfactant in the PBS buffer were prepared. Firstly, CrEL was dissolved in short chain alcohol-EtOH as a co-surfactant for the effective formation and stability of a nano-emulsion [27]. As a result, the cremophor emulsion with the core-shell micelles type [39] was obtained. The morphology of these micelles is typical of polymeric detergents, among them, CrEL. The architecture of such a micelle is based on the shell composed of a brush consisting of the hydrophilic polymeric part of CrEL and on a collapsed core of the hydrophobic chains of the detergent. Next, chl *a* at the molar concentration of 10^−5^ M was solubilized in the CrEL-PBS samples. In order to determine the level of chl *a* solubilization in different CrEL amounts, the fluorescence emission spectra were measured. As can be seen in Figure 2A, the intensity of chl *a* fluorescence emission detected at 675 nm increases along with surfactant concentration reaching the maximum upon 5 wt% CrEL (0.02 M) in the water medium.

**Figure 3:**
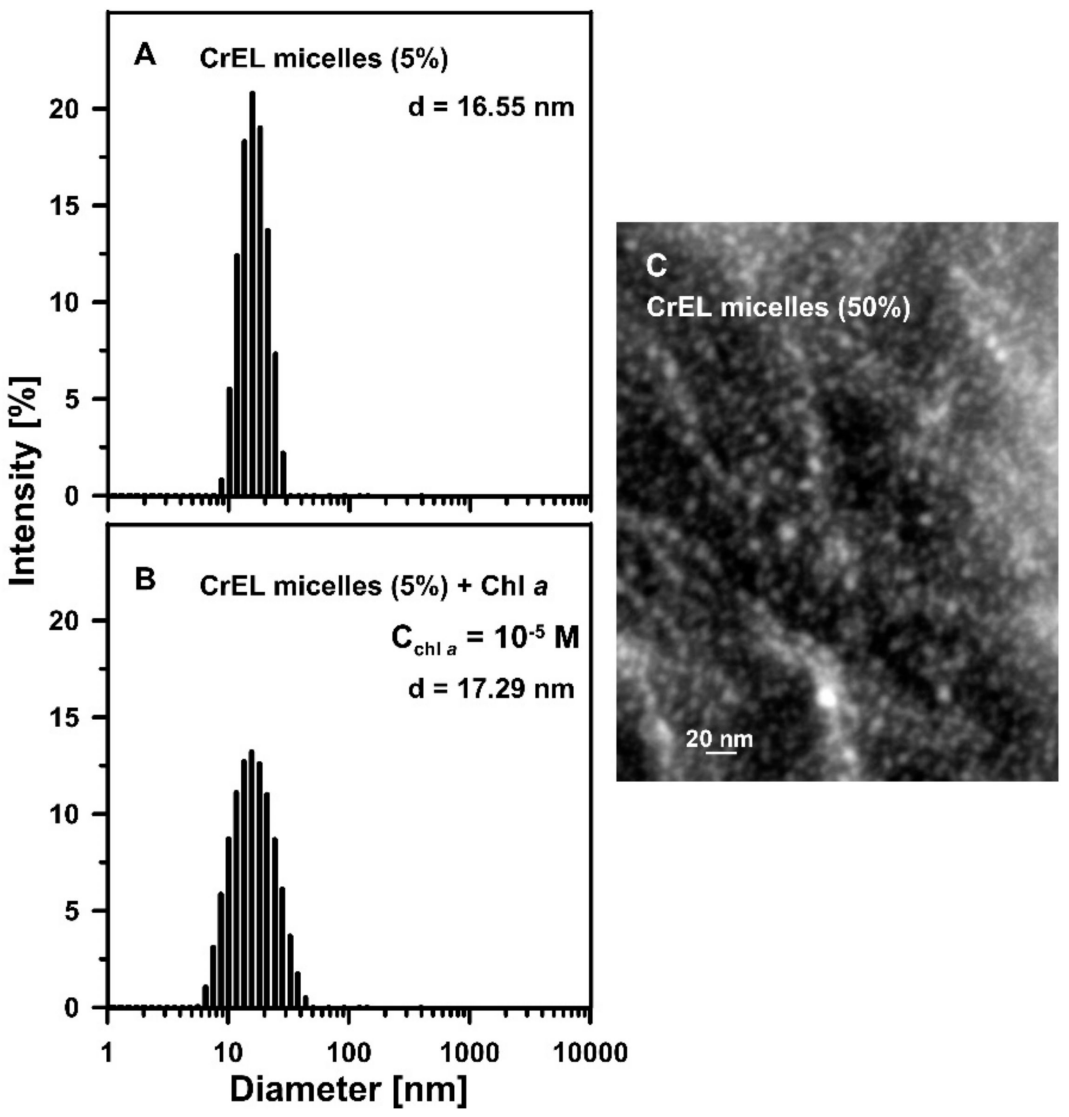
Cremophor micelles size distribution-histograms. (**A**): PBS buffer containing 5wt% CrEL (CrEL micelles (5%), 5% cremophor nano-emulsion). (**B)**: 5% cremophor nano-emulsion mixed with chl *a* at the molar concentration of 10^−5^ M (CrEL micelles (5%)+chl *a*). Histograms were obtained using the dynamic light scattering method. The representative sizes of nano-micelles are included in the figure (obtained from the presented histograms, numbers represents mean values obtained by replicate measurements of the same sample, n = 3 technical replicates). The experiment was repeated three times (biological replicates). (**C**): the Cryo-SEM image of cremophor nano-emulsion. For better visualization of nano-micelles CrEL was suspended in the PBS buffer at 50wt% concentration. Scale bar = 20 nm.

This increase in the fluorescence emission intensity points out to the chl *a* monomerization caused by pigment molecules transfer from the aqueous medium to the hydrophobic core of CrEL micelles [40]. Using the dynamic light scattering method the typical size of CrEL micelles in the 5wt% emulsion with or without chl *a* molecules was determined to be about 17 nm (Figure 3).

Thus, the co-surfactant and chl *a* molecules spontaneously self-organizing in the aqueous medium can be termed SNEDDS (self-nanoemulsifying drug delivery system) [41]. Such a system is known as a good hydrophobic drugs carrier [42,43,44,45,46]. Monomerization of chl *a* in SNEEDS is expected to improve its bioavailability in the potential gastrointestinal treatment. For comparison, chl *a* was also dissolved in different wt% concentrations of EtOH in the PBS buffer. EtOH is an important organic solvent extensively used in food, pharmaceutical and cosmetics industries, so it may also be applied in chl medical or food formulations. Figure 2B presents that chl *a* at 10^−5^ M concentration solvates only at above 50wt% EtOH in the PBS buffer. Thus, chl *a* dissolves more efficiently even in the aqueous medium containing low-level CrEL than in an ethanol-water solution. Based on the knowledge that an ethanolic solution above 15% is toxic for human health [47], encapsulation of chl *a* into the CrEL nano-micelles seems to be promising and justified.

**Figure 4:**
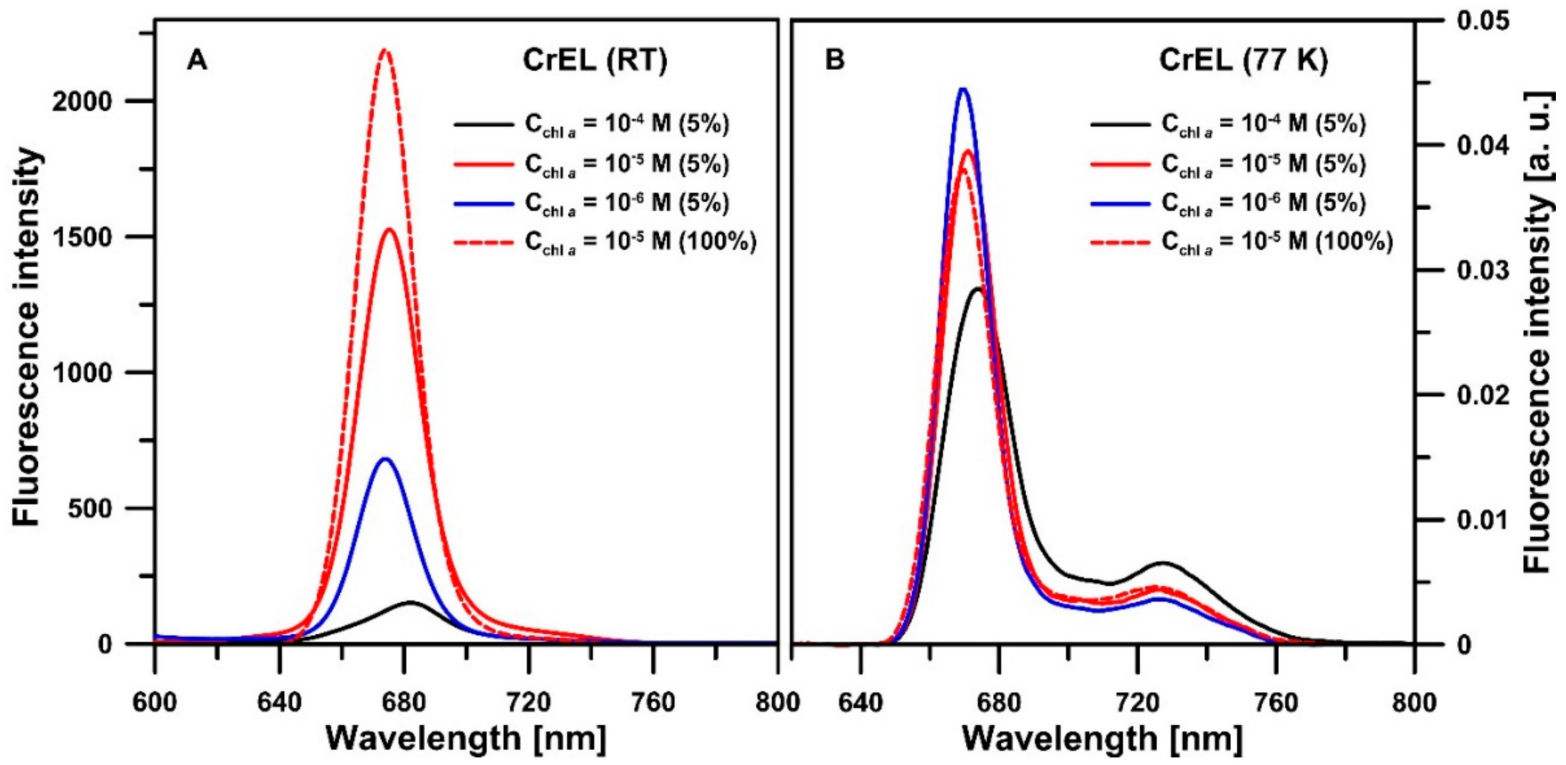
Room temperature and 77 K fluorescence emission spectra of chlorophyll *a* dissolved in the cremophor nano-emulsion. The molar concentrations of chl *a* in the samples were: 10^−4^ M (black solid line), 10^−5^ M (red solid and dashed line) and 10^−6^ M (blue solid line), respectively. Chl *a* was suspended in the PBS buffer containing 5 wt% CrEL (CrEL, 5% cremophor nano-emulsion). **A**: the spectra measured at room temperature, **B**: the spectra measured at 77 K. The 77 K fluorescence emission spectra were normalized to get the same area beneath each spectrum. The excitation wavelength was at 434 nm. In both panels the spectrum of chl *a* dissolved in CrEL (100%) was included.

The question ‘What is the largest amount of chl *a* that is still monomerized at such concentrations (5wt%) of the surfactant?’ is also addressed in this paper. Figure 4 presents the fluorescence emission spectra of chl *a* in different molar concentrations measured at room temperature (Figure 4A) and at 77 K (Figure 4B).

The excitation wavelength was set at 434 nm. The shape and position of the bands of chl *a* fluorescence emission spectrum provide important information about molecular organization of the pigment because aggregation/disaggregation of chl *a* affects the electronic structure of its macrocycles. Chl *a* monomer is characterized by intensive fluorescence but chl *a* dimers or larger aggregates are strongly fluorescence quenching molecular forms [48,49]. At 10^−4^ M, a decrease in chl *a* fluorescence emission intensity (fluorescence quenching) and the shift of the main band at 682 nm are observed, which can be due to high pigment concentration accompanied by excitonic interactions between the neighbouring molecules. Secondly, this result may point out to the poor solubility of chl *a* in such amount at low wt% concentration of detergent-emulsion. It cannot be excluded either that a decrease in chl *a* fluorescence emission intensity and the redshift of the main band may result from fluorescence reabsorption induced by a high chl *a* content followed by the secondary emission. However, intensive fluorescence emission at 675 nm of chl *a* at 10^−5^ M and 10^−6^ M indicated strong pigment monomerization at these molar concentrations. In order to confirm chl *a* molecular organization (in different molar concentrations) in the studied nano-micelles the spectra of fluorescence emission at 77 K were registered and normalized to get the same area beneath each spectrum. At low temperature, it is possible to observe fluorescence emission not only from monomers, as it is at room temperature, but also from such molecular oligomeric forms as dimers and aggregates. The quantum yield of fluorescence of aggregates is very low due to the vibrational relaxation mode. Lowering the sample temperatures increases fluorescence intensity and sharpens spectral bands which results from the loss of intramolecular vibrations. It is known that the band centered at about 670 nm is assigned to chl *a* monomers whereas the bands located at the longer wavelengths (≥700 nm) are typical of chl *a* dimers and aggregates [35,50,51]. Taking the above into consideration, it can be concluded that encapsulation of chl *a* in CrEL nano-micelles at 10^−5^ M and 10^−6^ M concentrations resulted in the efficient monomerization of the pigment. However, the band located at ~730 nm should be noted. It is visible in all the samples and its intensity increases along with chl *a* concentration, thus, it can be concluded that this band arises from the chl *a* aggregates, most likely presented in an aqueous phase of the nano-emulsion. The fact that the monomerization level of chl *a* (10^−5^ M) in the CrEL aqueous medium is the same as in 100% detergent is a satisfactory result. The 77 K fluorescence emission spectrum of chl *a* (10^−5^ M) in the 5 wt% CrEL aqueous medium has the same intensity and maximum position as that of chl *a* dissolved at this concentration in 100% CrEL. Thus, for further study, the 10^−5^ M concentration of chl *a* was selected as the largest pigment amount that is efficiently solubilized in the nano-emulsion used by us. This is a promising result, because Dentuto et al. [52] evidenced that such a concentration of chl *a* is non-toxic for human T lymphocyte cells.

**Figure 5:**
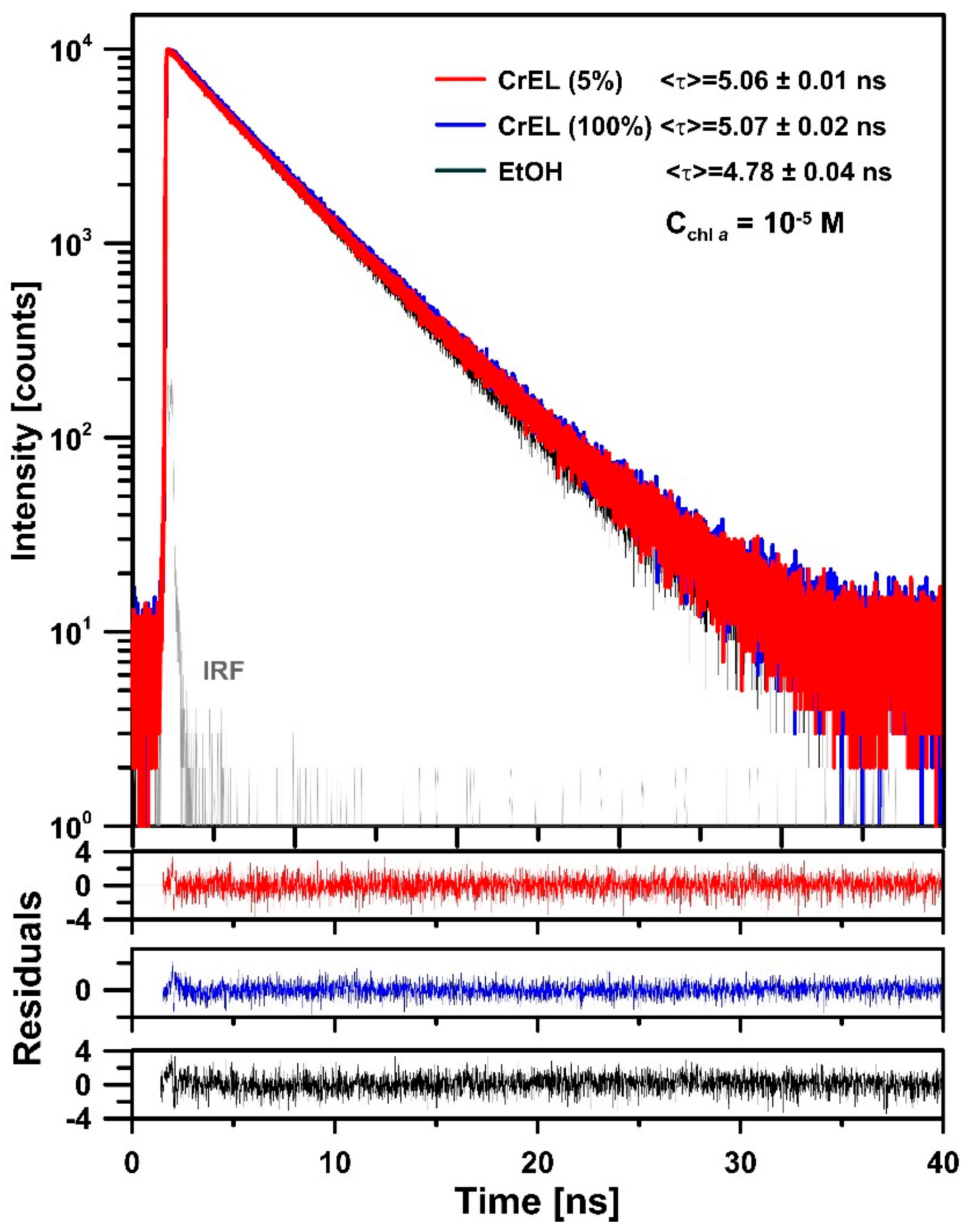
Fluorescence decay traces of emission of chlorophyll *a* dissolved in Cremophor EL, 96% ethanol or cremophor nano-emulsion. The molar concentration of chl *a* was 10^−5^ M. The excitation and detection wavelength were set at 405 nm and 670 nm, respectively. The fluorescence intensity decays were analyzed by reconvolution with the instrument response function (IRF, grey line) and analyzed as a sum of exponential terms. The quality of the fit was judged by the χ^2^ value (1.00-–1.03). The typical fluorescence lifetime decays, intensity-weighted average lifetime <τ> values (mean ± SD) and residuals of the curve fit are displayed in the figure. The fluorescence decay traces were registered at room temperature.

Molecular organization of chl *a* also affects its fluorescence decay kinetics. It is known that the averaged fluorescence lifetime of chl *a* depends on the solvent medium and concentration of the pigment. In vivo, when chl *a* is embedded into the protein bed of photosynthetic complexes in the thylakoid membrane, its intensity averaged fluorescence lifetime is about 2 ns [53]. The value of this parameter increases for the isolated trimeric (3.5 ns) and monomeric (2.7 ns) forms of the LHCII antenna complex [19]. The chl *a* fluorescence lifetime is the longest for the pigment monomeric form in diluted solutions ranging from 5 to 6 ns [15,32,33,49]. When the chl *a* molecules aggregate, the averaged fluorescence lifetime shortens significantly [32,49]. In Figure 5 the fluorescence decay kinetics of chl *a* (10^−5^ M) encapsulated in 5wt% CrEL nano-emulsion and dissolved in 100% CrEL or 96% EtOH is presented.

The values of intensity averaged lifetime of chl *a* fluorescence are τ = 5.06 ± 0.01 ns, τ = 5.07 ± 0.02 ns and τ = 4.78 ± 0.04 ns, respectively. The analysis has shown double (100% CrEL) or triple-exponential decay kinetics (5 wt% CrEL nano-emulsion and 96% EtOH) indicating the presence of different excited molecular species in the samples. The fluorescence decay kinetics were fitted with the components characterized by the following lifetimes: τ_1_ = 5.7 ns, τ_2_ = 3.7 ns and τ_3_ = 0.5 ns in the case of chl *a* suspended in 5 wt% CrEL in the PBS buffer (5% cremophor nano-emulsion (CrEL (5%)), red line, Figure 5), τ_1_ = 5.5 ns, τ_2_ = 3.3 ns in the case of chl *a* dissolved in CrEL (CrEL (100%), blue line, Figure 5), and τ_1_ = 4.9 ns, τ_2_ = 3.0 ns and τ_3_ = 0.2 ns in the case of chl *a* dissolved in 96% EtOH (EtOH, black line, Figure 5). The longest lifetime component (4.9–5.7 ns) can be attributed to chl *a* monomer [54,55]. The ~3 ns component may be assigned to dimers of chl *a* [55,56]. The fastest lifetime component ranging from 0.2 to 0.5 ns points out to the pigment tendency to form aggregates [54,55]. However, the value of the amplitude of the long lifetime component is on a fine level of 70.6 ± 0.4%, 78.9 ± 0.8% and 91.8 ± 1.9% in the case of nano-emulsion, 100% CrEL and EtOH, respectively, while the shortest lifetime component intensity is only 0.80 ± 0.05% (5wt% CrEL) and 0.50 ± 0.04% (EtOH) of the fluorescence signal. This component is not present in the case of chl *a* dissolved in 100% CrEL. These findings confirm that chl *a* encapsulation into the CrEL nano-emulsion is a potential powerful tool for chl *a* monomerization in an aqueous solution.

**Figure 6:**
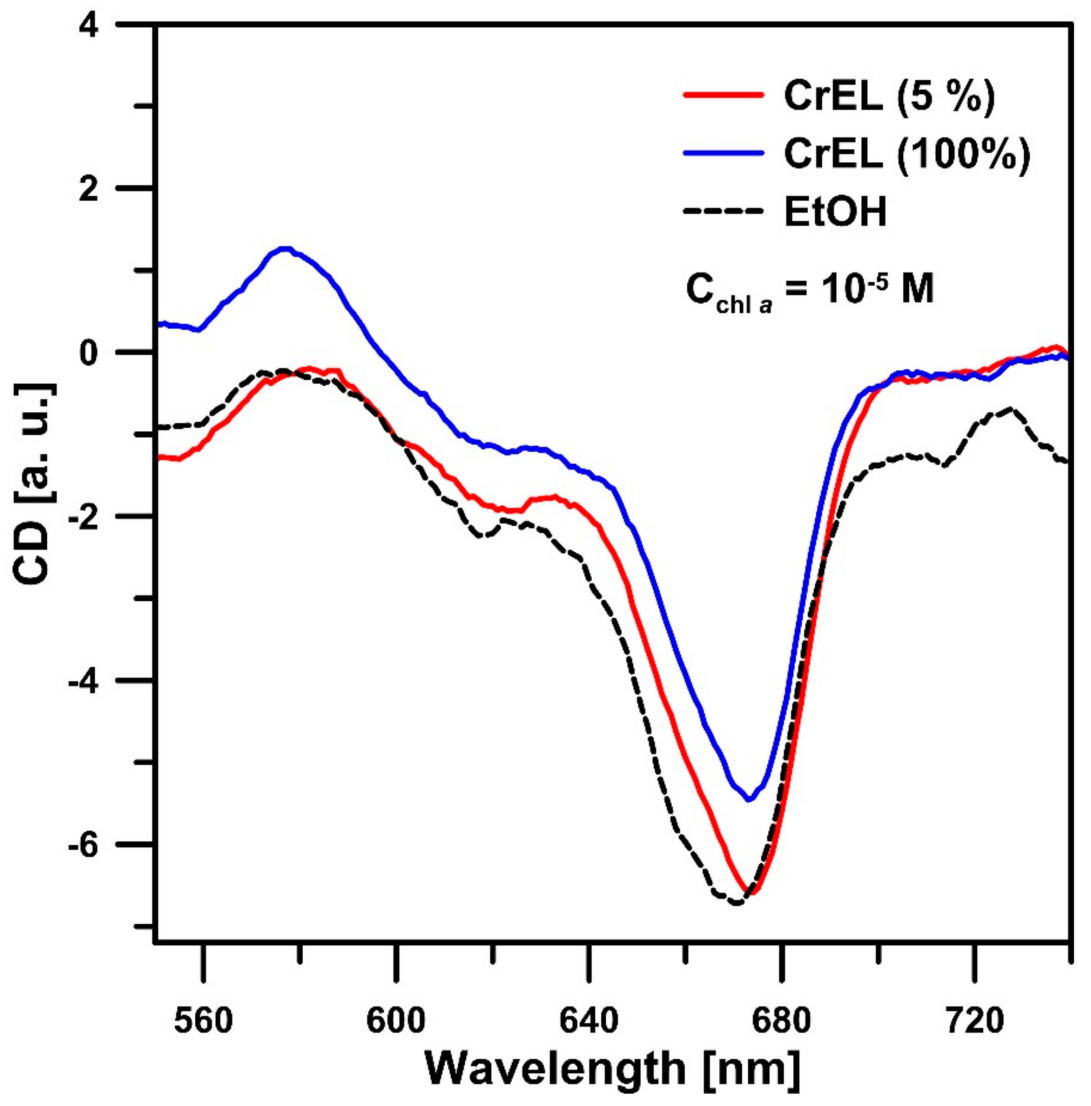
CD spectra in the red region of chlorophyll *a* dissolved in Cremophor EL, 96% ethanol or suspended in the cremophor nano-emulsion. Chl *a* at the molar concentration of 10^−5^ M was suspended in the PBS buffer containing 5 wt% Cremophor EL (CrEL (5%), 5% cremophor nano-emulsion, red solid line), dissolved in CrEL (CrEL (100%), blue solid line) or 96% EtOH (EtOH, black dashed line).

Molecular organization of chl *a* in the 5wt% CrEL nano-emulsion and 100% CrEL or 96% EtOH has been also deduced from the circular dichroism (CD) spectra (Figure 6).

This is possible because of the relationship between the molecular structure of the pigment and its optical activity. In the solution, chl *a* exhibits a weaker CD signal in comparison to the pigments embedded into the protein bed [57]. Moreover, in the case of chl *a* monomer, CD signal results from intrinsic asymmetry of the molecule. Molecular aggregates generate characteristic CD spectrum shape (splitting into positive and negative bands) originating from the excitonic interactions between chromophores [51,57,58]. The Q spectral region of CD spectra presented in Figure 6 shows one characteristic negative band at 674 nm (for both cremophor samples) and 668 nm (for chl *a* dissolved in EtOH). The same behavior was exhibited by the spectrum of bacteriochlorophyll c monomer presented by Olson and Cox [58]. Based on this knowledge, our finding can be interpreted as a confirmation of chl *a* monomerization in the 5wt% CrEL nano-emulsion.

**Figure 7:**
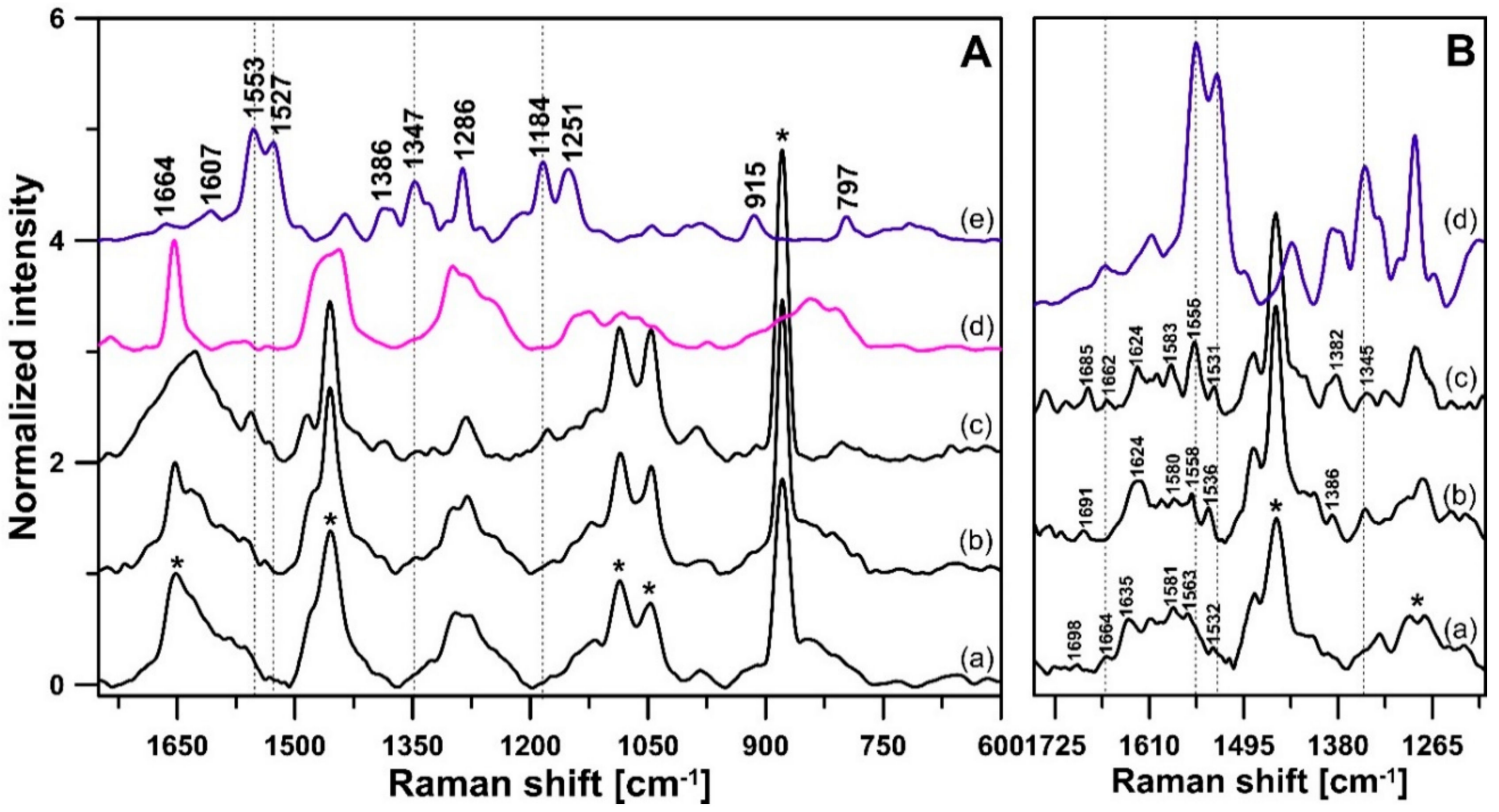
Resonance Raman spectra recorded for chlorophyll *a*, cremophor nano-emulsion and the mixtures of nano-emulsion-chlorophyll *a*. (**A**): Chl *a* in EtOH (blue line, (e)), CrEL magenta line, (d)), and chl *a* in PBS buffer containing 0.5 (a), 5 (b), and 50wt% (c) CrEL (black lines) at the concentration of 10^−5^ M. (**B**): RR spectra of Chl *a* in EtOH (d), and chl *a* in the PBS buffer containing 0.5 (a), 5 (b), and 50 wt% (c) CrEL after subtraction of the exact concentration of CrEL in the aqueous medium. The Raman bands typical of both CrEL and EtOH were marked with asterisks.

The resonance Raman (RR) spectra of chl *a* dissolved in EtOH and in the CrEL nano-emulsion system (PBS buffer containing 0.5, 5, and 50wt% CrEL, respectively) and excited by the 457 nm laser line are presented in Figure 7A.

The Raman bands in the spectrum of chl *a* in EtOH, recorded under pre-resonant conditions, arise from the normal vibrations of the conjugated system, i.e., the macrocycle and the peripheral vinyl and the 9-keto carbonyl group. Its spectral feature is in good agreement with those measured in polar solvents and reported somewhere else [59,60,61]. The conjugated aromatic pyrrole rings coordinated with the central magnesium(II) ion give characteristic Raman bands in the range of 1670–1600 cm^−1^ (weak), at 1553 cm^−1^ (strong), and in the range of 1529–1527 cm^−1^ (medium) that indicates that the Mg atom of chl *a* is 5-coordinated [62]. It was shown that the frequency of the C=O stretching group as well as the number of bands in the carbonyl region were used to distinguish the aggregation state of chl [59]. Contribution of the predominant band at 1653 cm^−1^, arising from the C=O group, observed for the CrEL solution (Figure 7A(d)), may make the interpretation of the data in the spectral carbonyl region complicated. Therefore, the spectra of CrEL exact concentration of CrEL in the aqueous medium s are subtracted from the spectra of chl *a* in these solutions (Figure 7B(a–c)). As can be seen in Figure 7A, correlations between the RR spectrum of chl *a* in EtOH, and in the CrEL mixtures are found. However, all of the observed bands in the 1520–1560 cm^−1^ region (Figure 7A(a–d)) are shifted towards higher frequencies as compared to those recorded in the case of chl *a* in EtOH. To more detailed analysis, the spectra of chl *a* in the CrEL nano-emulsion, after subtraction of the pure CrEL in the aqueous medium in the range from 1250 to 1730 cm^−1^ are presented after in Figure 7B. The stretching mode of the C=O group of monomeric chl *a* (pigment dissolved in EtOH) appears as one separated band at 1664 cm^−1^ (Figure 7B; (d)), while the spectra of CrEL nano-emulsion with chl *a* display two components in the carbonyl region. The band observed at around 1698 cm^−1^ in the spectra of chl *a* in the 50wt% CrEL solution is downshifted with respect to the corresponding band of the samples with the lower detergent amount. This band is reported to be not disturbed by other vibrations, and its presence in the high-wavenumber region indicates the absence of intermolecular interaction [63,64,65]. Therefore, such a downshift observed here could be related to H-bond formulation between chl *a* molecules and the medium. Additionally, the latter component from this region, assigned to the stretching modes of the conjugated carbonyls [59], which is sensitive to the nature and strength of intermolecular interactions assumed by the C=O groups, has a low intensity at about 1660 cm^−1^. Thus, the lack of bands arising from the self-associated or hydrated chl *a* molecules allows to suppose that essentially monomeric chl *a* exists in the CrEL nano-emulsion system. Comparing the RR spectra of chl *a* in the CrEL system with that obtained for chl *a* in EtOH, a few supplementary bands were observed in the region of 1630–1520 cm^−1^. The first additional band located at 1624 cm^−1^ has been assigned to a conjugated vinyl on the chlorine ring [66]. Interestingly, its appearance in the spectra of chl *a* in a low amount (5wt%) of CrEL is accompanied by the downshift of the C=O stretching mode from 1698 cm^−1^ to 1685 cm^−1^. On the other hand, a solvent effect was found for the latter band near 1580 cm^−1^ assigned to the ring-breathing mode. Among many bands observed in this part of RR spectra there are minor differences compared with the monomeric form of chl *a* in ethanol, and they could be related to the interactions between chlorophyll and the detergent molecules. Similar results were found in the 1200–1500 cm^−1^ region, particularly for the spectra of chl *a* dissolved in a low amount (1 wt%, data not shown and 5 wt%, Figure 7B(b)) of CrEL. Therefore, it is supposed that essentially the monomeric forms of chl *a* exist in the CrEL system.

**Figure 8:**
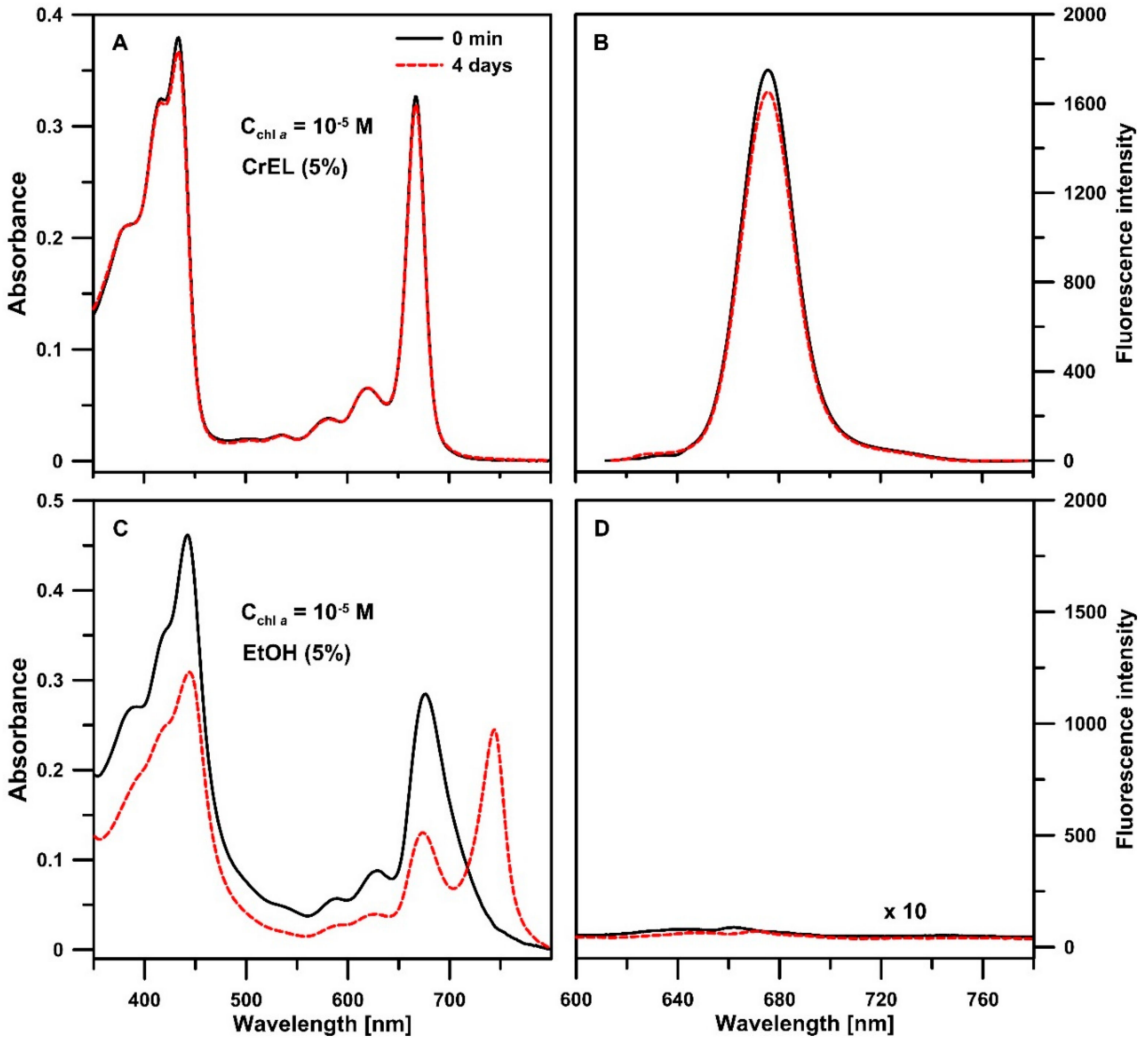
Electronic absorption (**A,C**) and fluorescence emission (**B,D**) spectra of chlorophyll *a* dissolved in 5 wt% Cremophor EL or 5% ethanol-PBS mixture. Absorption spectra suspended in CrEL (5wt%) or 5% EtOH-PBS solution. The spectra were registered directly after the samples preparation (black solid line) and after four days (red dashed line). The emission spectra were registered with excitation in the Soret band maximum. The molar concentration of chl *a* in the samples was 10^−5^ M. The samples were kept in the oxygenic condition and in the darkness. The spectra were measured at room temperature.

Finally, a crucial feature of 5wt% CrEL nano-emulsion system is to provide stability of chl *a* monomeric form. As it is shown in Figure 8, the spectral shape of light absorption (A) and fluorescence emission (B) of chl *a* in the 5wt% CrEL nano-emulsion, at concentration of 10^−5^ M, did not change after four days of preparing the samples. This result is interesting concerning the market of food colorants. The instability of pure chl in the colorant product related to the food processing causes a drastic change in color from green to brown [67,68]. The usage of chl *a* dispersed in CrEL might limit the reduction or even withdrawal of the original green color of product that is desired by the food industry.

Simultaneously, the intensity of absorption at 666 nm and fluorescence emission at 675 nm decreased only by 11% and 6%, respectively. On the basis of the position of the absorption bands maximum (442 nm and 676 nm, Figure 8C) and loss of fluorescence signal (Figure 8D), it can be concluded that chl *a* aggregates in 5% EtOH-PBS mixture. Additionally, the aggregated molecular organization of chl *a* changed in four days. Similarly, previous studies have reported that the EtOH–water mixture changes chl *a* molecular organization [69]. Moreover, under illumination, monomeric chl *a* dissolved in absolute EtOH is stable only for a few hours [70].

## 4. Conclusions

In presented experiments, chl *a* was encapsulated into the CrEL nano-emulsion system. Spontaneously self-organizing Cremophor micelles size was determined to be about 17 nm. Such a system with chl *a* was characterized by molecular spectroscopy techniques (electronic absorption, steady-state and time-resolved fluorescence, circular dichroism and Raman spectroscopy). On the base of the fluorescence emission spectra we concluded that chl *a* dissolves more efficiently in the aqueous medium containing low-level CrEL (5 wt%) as compared to ethanolic solution. Moreover, Raman spectra have shown that molecular organization of chl *a* in CrEL aqueous solution is mainly monomeric. The study by means of 77 K fluorescence emission exhibited that the monomerization level of chl *a* (at 10^−5^ M) in 5 wt% emulsion is the same as in the 100% detergent. It was also demonstrated that CrEL nano-micelles system stabilizes chl *a* monomers. In summary, application of CrEL nano-emulsion system to chl *a* dissolution in water medium could increase its practical application in medicine (e.g., in photodynamic therapy), food industries (e.g., as food colorant or dietary supplement) or modeling of well-defined molecular artificial system.
